# Local vs general anaesthesia in the development of neurosensory disturbances after mandibular third molars extraction: A retrospective study of 534 cases

**DOI:** 10.4317/medoral.21238

**Published:** 2016-10-01

**Authors:** Fulvia Costantinides, Matteo Biasotto, Michele Maglione, Roberto Di Lenarda

**Affiliations:** 1 Unit of Oral Surgery, School of Dental Sciences, Department of Medical, Surgical and Health Sciences, University of Trieste, Italy

## Abstract

**Background:**

The choice of the anaesthetic modality is one of the primary steps during planning of third molar surgery. The aim of the present study was to compare the risk of developing neurological injures of the inferior alveolar nerve (IAN) and lingual nerve (LN) in patients treated for wisdom teeth removal under general anaesthesia (GA) with a group treated under local anaesthesia (LA).

**Material and Methods:**

This is an observational retrospective, unicentric study; between September 2013 and September 2014, 534 patients underwent third molar surgery, 194 (36,3%) under GA and 340 (63,7%) under LA by the same oral surgeon. Differences in the incidence of IAN and LN injures between groups have been statistically analyzed with Fisher exact test and estimated odd ratio for development of such complications has been calculated.

**Results:**

In GA patients the incidence of IAN and LN injures was 4.6% and 2.1%, respectively while in the LA group it was and 0.3% and 0%, respectively. A significant difference in IAN and LN involvement between groups was observed (IAN lesion: Fisher exact test, *p*<0.001; LN lesions: Fisher exact test, *p*<0.05). The estimated odd ratio for development of IAN injures after GA was 16.49 (95% CI: 2.07-131.19) and was not calculable for LN injures because no cases were observed in the LA group.

**Conclusions:**

Since GA is a perioperative variable that seems to significantly increase the risk of developing IAN and LN lesions, when treating patients that request GA, they must be adequately informed that an higher incidence of post-surgical sensory disturbances is expected.

**Key words:**Third molars, general anaesthesia, local anaesthesia, inferior alveolar nerve, lingual nerve.

## Introduction

Third molars extraction represents a frequent surgical procedure rich of operative variables that the oral and maxillofacial surgeon has carefully to take into consideration to perform an accurate preoperative evaluation. The clinician has to establish the role of the third molar in the oral economy and the presence of some pathologic conditions associated to the wisdom tooth to eventually justify the extraction. Afterwards, the operator has to evaluate the patient’s variables (age, gender, ethnicity, systemic pathologies), the tooth’s characteristics (morphology, deep of impaction and angulation, anatomic relationship with surrounding tissues), the radiographic findings (mandibular canal course, radiolucent/radiopaque lesions associated to the tooth, width of the follicle, roots development).

Furthermore, the planning of the third molar surgery needs the choice of the anaesthetic modality (local anaesthesia or general anaesthesia). Nowadays, apart the cases of atypical position of the third molar or displacements in anatomical spaces, considering that the operative times are generally prolonged and that the risk of postoperative morbidity increases in case of general anaesthesia, the choice of this modality depends fundamentally from a patient’s preference.

Among postoperative complications, neurological injures of the inferior alveolar (IAN) and lingual (LN) nerves represent a rare event but with a heavy impact on the patient’s quality of life and with possible medico-legal contentious for the surgeon. A previous report evaluated the association between general anaesthesia and neurological involvement in 183 patients with a prevalence of 5,8% for IAN and 0,3% for NL (1). In cases of local anaesthesia, IAN injury ranges from 0.6% to 5.8% and studies have shown the incidence of LN injuries to be variable and depending on a number of factors including techniques used, with rates between 0.2% and 1.6% (2-6).

The aim of the present study is to compare the risk to developing neurological injures of the IAN and LN in a group of patients treated for wisdom teeth removal under general anaesthesia (GA) with a group treated under local anaesthesia (LA) by the same operator. The null hypothesis is that the anaesthetic modality does not influence the outcome “sensory disturbances” of the labial or lingual region.

## Material and Methods

From a sample of 2044 patients consecutively treated for dental surgery at our department between September 2013 and September 2014, 558 needed the extraction of the lower wisdom teeth and were included in the study. No exclusion criteria were adopted. An informed written consent was obtained from each patient to use clinical data for the research that was conducted in agreement with the guidelines of the Helsinki Declaration as revised in 1975 and amended in October 2003. The study has been approved by the local ethical commmettee.

- Radiographic evaluation and surgical procedures

All mandibular third molars were radiographycally evaluated by orthopantomogarphy (OPT) before surgery. When a real contact between alveolar inferior canal and third molar roots was suspected due to the presence of Rood’s signs, a computed tomography (TC) or a cone-beam computed tomography (CBCT) was performed.

The same operator (M.B.) proceeded with the extractions under LA or GA with standardized surgical instruments and procedures. For each patient a dose of 2 ml mepivacain 20mg/ml/adrenaline 1:100.000 was infiltrated in correspondence of the Spix spine and 1 ml in the vestibular oral mucosa. When necessary, for totally or partially impacted molars, a buccal total thickness trapezoidal flap was raised. Accurate periosteal elevation was made, particularly on lingual zone. Lingual flap was protected, not retracted, using a Prichard elevator during all the surgical procedures (ostectomy, crown sectioning and luxation) to preserve soft lingual tissues and consequently the lingual nerve that often is localized near inferior third molar few millimetres distally and lower respect to second molar and levelled with or superior to the crest of the lingual plate. Ostectomy and tooth sectioning phases were performed using diamond or Allport burs inserted on low speed handpiece (30.000 rotations/minute), always irrigated with sterile physiological solution. Silk sutures were used for the closure of the gingival tissues. For all patients, independently of the anaesthetic modality, antibiotic and anti-inflammatory medication was prescribed when necessary (usually amoxicillin 2 g or clarithromycin 500 mg, two times a day for 5 days and ibuprofen 600 mg, 2 times a day for 3 days), with 0.2 % chlorexidine rinses 2 times a day for 7 days.

- Follow-up

Postoperative assessment for hypoesthesia, paraesthesia, anaesthesia was done by two expert oral surgeons (M.M. and M.B.) after one week at the time of suture removal by questioning about tongue, chin, and lip sensibility and performing neurosensory tests like 2-point discrimination, pinprick, and light touch. Patients with neurosensory disturbance were followed up each month for six months. The presence of permanent or temporary neurological IAN/LN injures occurring after extraction was assessed, classifying lesions as temporary if resolved in 6 months.

- Data collection

The following data were collected from the clinical records of all patients: classification according to Pell and Gregory subdivision, degree of inclusion (erupted, mucosal retention or bone retention), pathologic conditions that motivated tooth extraction (malposition with recurrent pericoronaritis, chronic periodontitis, mucosal traumatism, caries, involvement of the second molar, abscess, orthodontic request, cysts), type of anaesthetic modality (GA or LA), surgical technique (flap preparation, ostectomy and tooth sectioning), events of IAN/LN temporary or permanent lesion. For patients who needed bilateral removal, only the first tooth extracted was considered for analysis.

- Statystical analysis

The SPSS software, version 15.0 (SPSS® Inc., Chicago, Illinois, USA) was used for the statistical analysis.

Non-parametric methods were chosen after testing the normality of the data using a Shapiro–Wilk test and the equality of variance among the datasets using a Levene test, with the exception of the ages of the groups.

Equality of groups by age and sex was tested by a Student t-test and a chi squared test.

The Fisher exact test was used to assess the significance of the difference for all the other variables between groups. The odds ratio of developing neurological lesions was estimated (calculated as the ratio of the odds between anaesthetic modalities).

Results

On a total of 558 patients, 24 did not return for the follow-up visits after extraction and were not considered for the study. A final sample of 534 patients was obtained. 283 subjects were male (53%) and 251 were female (47%) with a sex ratio M/F of 1.1/1 and an age range 14-89 years, mean 41.3, standard deviation (SD) ±17.8. 194 patients (36,3%) were treated under general anaesthesia (GA group), 92 were males (47.4%) and 102 were females (52.6%) with a sex ratio M/F of 0.9/1 and an age range 14-83, mean 33.8, SD ±15.2; 340 patients (63.7%) were treated under local anaesthesia (LA group), 191 were males (56.2%) and 149 were females (43.8%) with a sex ratio M/F of 1.3/1 and an age range 17-89 years, mean 45.5, SD ±17.8.

The mean of the ages, the gender distribution and the subdivision of the avulsed teeth following the pre- and post- surgical variables are shown in  Table 1.

**Table 1:**
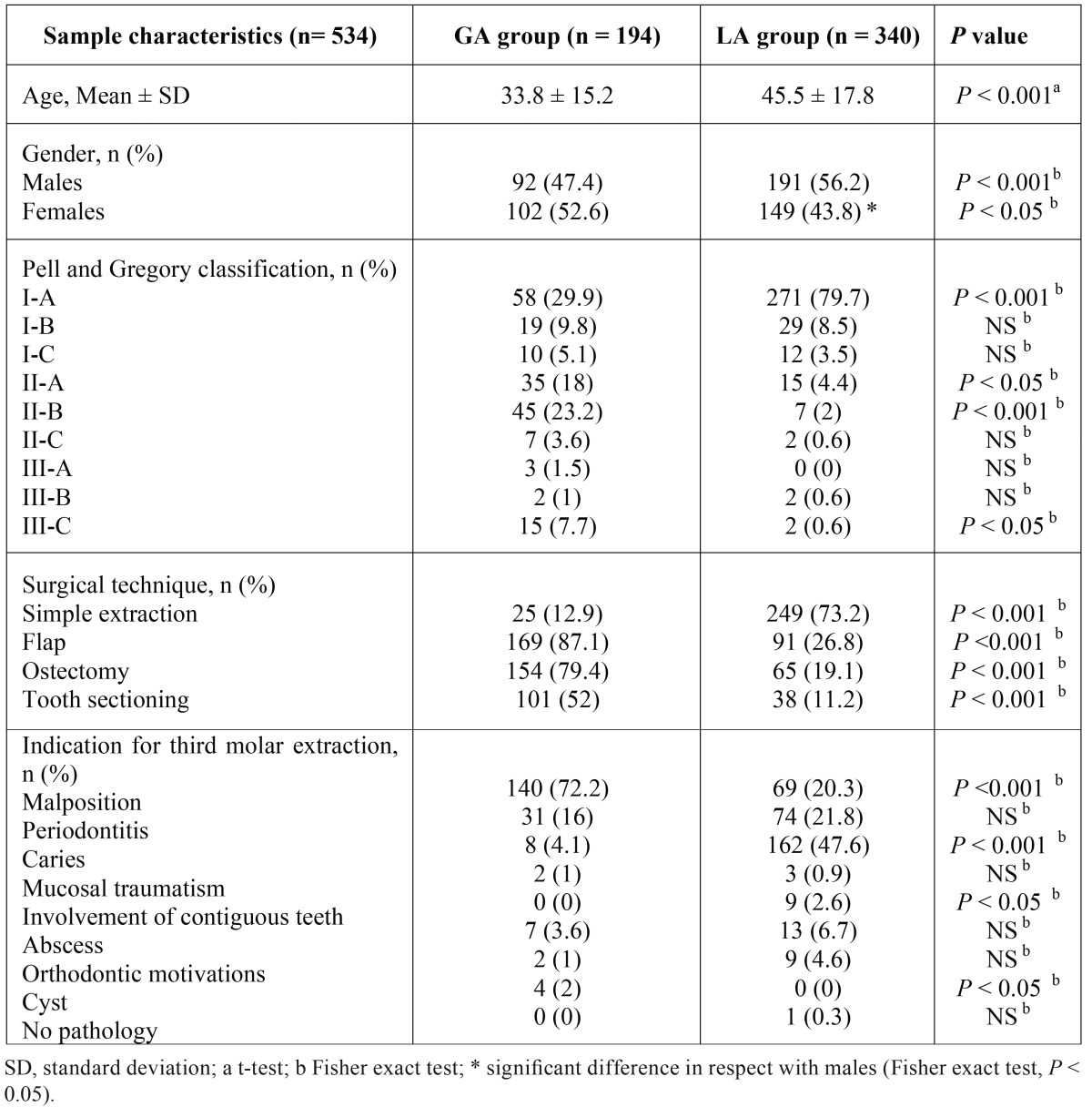
Distribution of the patients undergoing general anaesthesia (GA group) and local anaesthesia (LA group) in respect with age, gender, radiological classification, type of surgery and indication for tooth extraction.

Statistical analysis showed a significant difference in mean age between GA group and LA group (t-test, *P* < 0.001). A difference was also observed in gender distribution between groups (males: Fisher exact test, *P* < 0.001; females: Fisher exact test, *P* < 0.05). Moreover, a significant difference in males/females distribution was seen for LA group (Fisher exact test, *P* < 0.05). Regarding the radiological classification, a significant difference was observed for class I-A (Fisher exact test, *P* < 0.001), II-A (Fisher exact test, *P* < 0.05), II-B (Fisher exact test, *P* < 0.001), III-C (Fisher exact test, *P* < 0.05).

All the variables associated to the surgical technique showed a significant difference, with a preponderance of simple extractions in the LA group (Fisher exact test, *P* < 0.001).

Differences in the indications for third molar extraction were observed for malposition (Fisher exact test, *P* < 0.001), caries (Fisher exact test, *P* < 0.001), involvement of contiguous teeth (Fisher exact test, *P* < 0.05) and cyst (Fisher exact test, *P* < 0.05).

Cases with IAN and/or LN temporary or permanent injures are described in  Table 2.

**Table 2:**
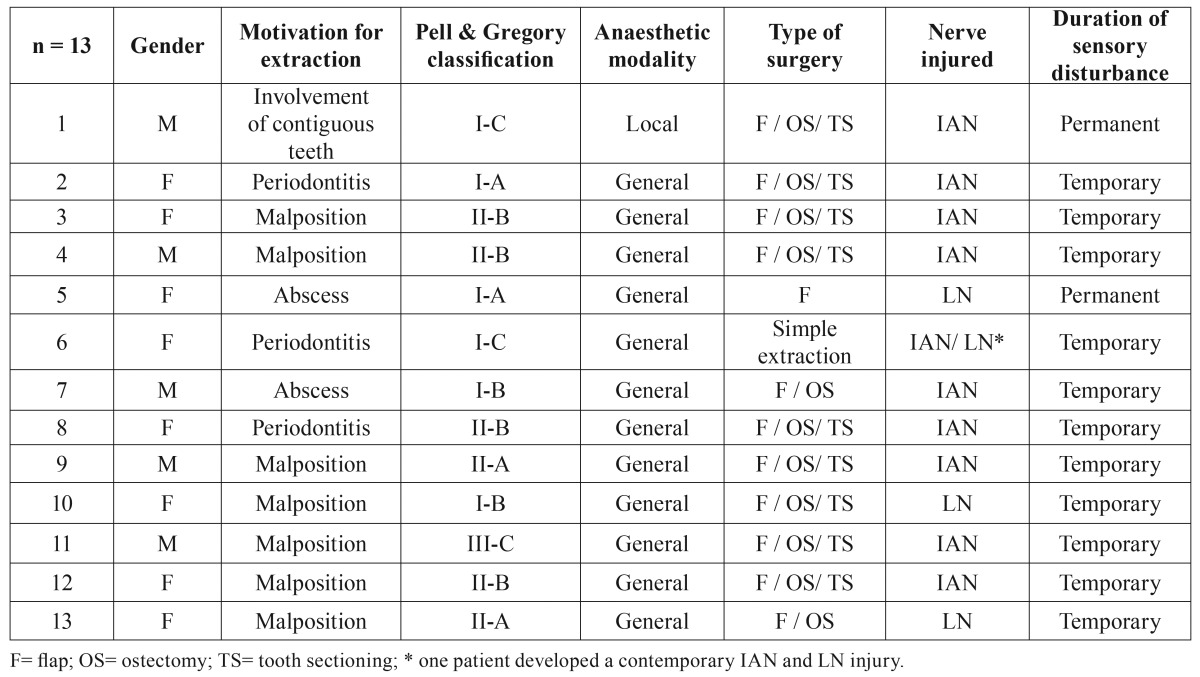
Clinical variables of patients affected by post-extractive sensory disturbance.

 Table 3 shows the incidence of sensory disturbances in the two groups. A significant difference was seen for both nerves between GA and LA groups (Fisher exact test, *P* < 0.001). The estimated odd ratio for development of IAN injures was 16.49 (95% confidence interval: 2.07-131.19) for molars extracted under GA and was not calculable for LN injures because no cases were observed in the LA group.

**Table 3:**
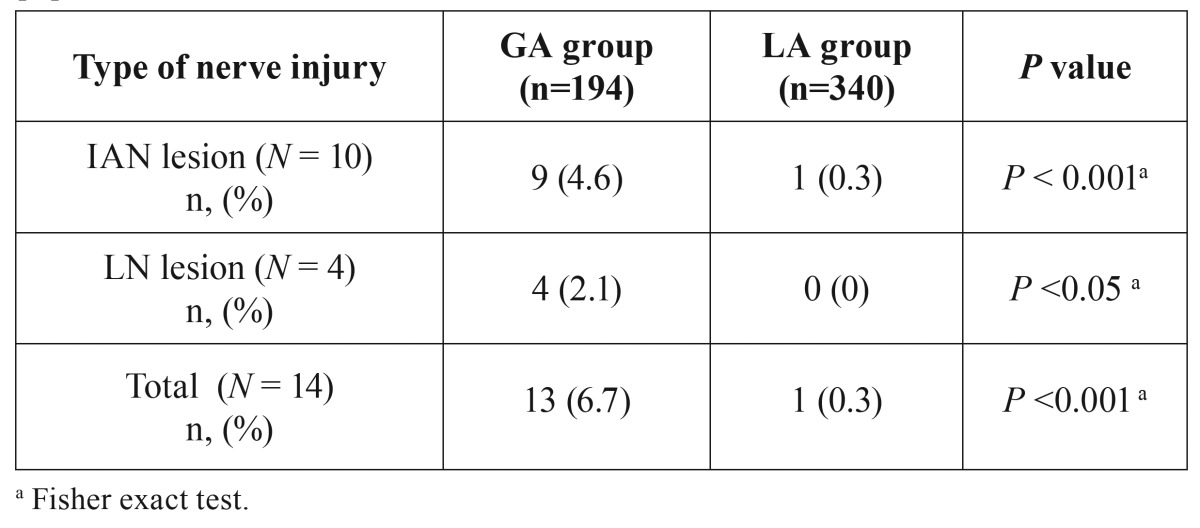
Incidence of temporary and permanent IAN and LN involvement in the study population.

**Figure 1:**
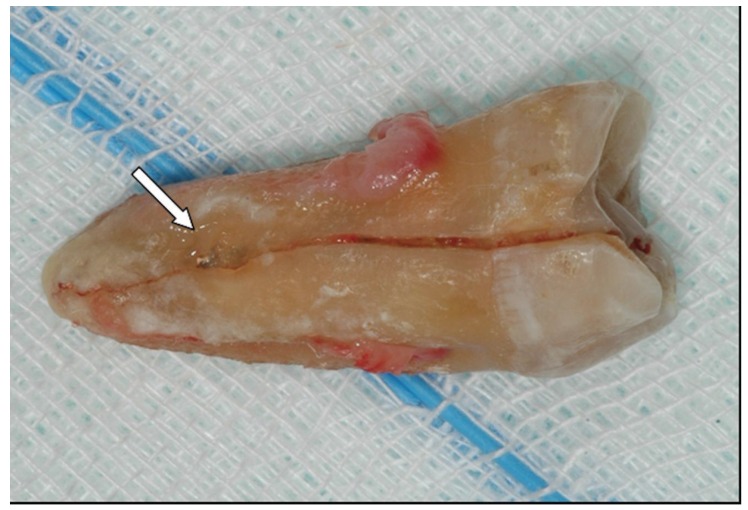
Mandibular right third molar extracted under local anaesthesia in a 49-years male who developed a NAI permanent paraesthesia. White arrow indicates the canal between fused roots were the IAN was entrapped.

On a total of 340 patients treated under LA, only one developed a IAN permanent paraesthesia. The patient was a 49-years male who needed the extraction of the inferior right third molar belonging to the class I-C and normally inclined. The extraction was indicated because of the presence of a deep periodontal pocket distally to the second molar caused by the third molar. The avulsion needed a flap preparation, the ostectomy of the vestibular bone wall and the tooth sectioning. The tooth fragments were re-assembled after the extraction showing a canal between the roots in which the NAI was entrapped (Fig. 1).

## Discussion

A series of clinical variables are correlated to an increased risk of neurological injures of IAN and LN after inferior third molar surgery. Some of these are patient- or tooth-dependent and are substantially unmodifiable (gender, ethnicity belonging, grade of impaction, angulation, roots development, pathology correlated to the tooth) others are operator- or technique-dependent and could be optimized to reduce the possibility of lesions (operator seniority, clinical instrumentation, surgical technique) (7). Among these last, the impact of anaesthetic modality on the clinical outcome has to be investigated to define if the risk of developing a temporary/permanent LN or IAN injury is influenced. Nonetheless, few researches focused the attention on this important clinical variable. Edwards *et al.* found that difficulty of surgery, patients’ anxiety levels, patients’ preferences, medical history and numbers of teeth to be removed are important predictors of choice of anaesthetic (8). Renton *et al.* 2001 studied the differences between patients who had undergone third molar removal under LA and GA. Authors found that, regardless of surgical difficulty, anaesthetic choice, in particular decision to extract under GA, are of important in terms of levels of morbidity, time of work and demands on primary care but they did not investigate the differences in terms of risk of neurological complications (9). Brann *et al.* found that nerve damage was five times more frequent when the extraction was performed under GA rather than LA (10). Conversely, Rehman *et al.* did not observe any significant relationship between nerve damage and anaesthetic modality although they found an increased risk of LN damage for unerupted teeth that requested a lingual retraction (11). Considering that, in their work, authors used a lingual retractor in 71.7 % of cases under GA and only in 32,1% of removals under LA, this could explain the increased neurological injures under GA even if not statistically significant. Hill *et al.* obtained the same results and did not find any statistical difference in the incidence of LN damage for lower third molar removal under LA and GA (12). The total rate of temporary nerve damage was under 5% in both groups with no cases of permanent lesions. However, authors underlined that in the GA group the adverse events rate per tooth was higher in the unilateral operations and that, in this group, the patients had recurrent episodes of pericoronaritis. This finding agrees with our previous study where an odds ratio of 6.86 was found for the variable “abscess” in correlation to the risk of post-surgical paraesthesia under GA (13).

The contrasting results regarding the influence of anaesthetic choice on the outcome “neurosensory disturbance”, could be biased by:

1- The indications followed to choose the GA.

2- The difficulty of the surgery for LA and GA groups.

The first point is still discussed. Edwards *et al.* remarked that in their clinical practice only those patients who require surgery under GA are listed for this and that LA or LA plus sedation are preferred when possible (8). Furthermore, they observed that, in UK, patients are referred unnecessarily for third molar surgery under GA because the family dentist often believes that the incidence of disease associated to unerupted or partially erupted tooth is much higher than is generally found. A clinical assessment before extraction is able to reduce the number of GA in a percentage of 15%. Also Rehman *et al.* found that the choice of anaesthetic modality is normally a result of patient decision (11).

Following the guidelines of the Royal College of Surgeons of England, GA is considered an appropriate method that may be needed for complex and longer procedures but that is correlated to higher risks in respect to LA. An emphasis was given to the possible fatal complications associated to the procedure so that all behavioural management and anxiety control have to be applied before proceeding with treatment under GA. Also guidelines of the National Institute of Clinical Excellence (NICE) for removal of wisdom teeth published in 2000, state that risks associated with the need for GA include rare and unpredictable death. The clinical guidelines published in 1997 (leaved unchanged in the 2004 revision) by the Faculty of the Royal College of Surgeons of England for third molars removal state that the anaesthesia selected depends upon a number of factors but without specifying them (14). Guidelines underline that only limited areas of discussion remainregarding third molar surgery and that main areas of variation in practice relate to removal vs retention and observation of pathology free impacted third molars and also to anaesthetic/analgesic/sedation modality. A research regarding the effect of the clinical guidelines on practice for extraction of lower third molars during the period 1997-2000 in UK, showed that the proportion of patients undergoing the surgery under GA decreased from 66% in 1997 to 54% in 2000 (15). This decrement has been related to patients’ choice, influence of the surgeon on patient, understanding of complications of GA, the availability of the facilities or the waiting list for GA. The report of Dunne *et al.* (16) took into consideration the effect of clinical guidelines of the Scottish Intercollegiate Guidelines Network (published in 2000) in Scottish oral and maxillofacial surgery units and found that patients treated under GA decreased slightly between 1995 and 2002 from 32% to 28% and that the choice of the GA was associated with significantly greater morbidity (particularly LN damage) (16). For this reason a precise information to the patients on anaesthetic options is mandatory.

It can be concluded that, actually, in literature, there are not well-established indications for choosing GA in third molar surgery although there is a general consensus regarding the increased co-morbidities associated to this anaesthetic modality.

The second point is strictly dependent for the first one. If the choice of the GA is based on the estimated difficulty of the surgery by the clinician, a direct consequence will be a inhomogeneous distribution of cases and a bias in the assessment of the influence of LA and GA on the neurological lesions. In fact, if the surgical difficulty is greater, an increased percentage of injures is expected. Conversely, if the anaesthetic regimen depended solely on the choice of the patient, it would get a better distribution of the surgical variables and a more reliable comparison. Brann *et al.* did not find any significant difference regarding surgical difficulty using the Wharfe score in the LA and GA groups (10). They observed that the incidence of nerve damage for teeth removed under GA was greater than five times the incidence of lesions for those removed under LA. Authors suggested that this findings could be the results of different variables: the supine position of the patients during GA, the increased extension of the mucoperiosteal flap and bone removal, the increased surgical force. Conversely, in their study Rehman *et al.* showed that, even if Wharfe and Vas scores were similar for cases performed under GA or LA, there was no significant difference in surgical difficulty between the two groups (11). These and other contrasting results suggest that more research is required to understand the real impact of the anaesthetic modality on the neurological damage so that the surgeon could make the choice based on evidence and inform adequately the patient.

In the present study, GA was explicitly requested by those patients that refused third molar extraction under LA (odontophobic), although the local and systemic complications associated to the procedure have been clarified before surgery. Furthermore, GA has been chosen by surgeon in four cases of cystectomy and in those patient who needed the contemporary extraction of the four third molars, when malposed and partially or totally included. None of the teeth were removed under GA if asymptomatic, while only one was extracted in the LA group (prosthetic necessities).

Although the GA regimen was substantially a patient’s choice, data showed that teeth extracted under GA were rarely normally erupted and were generally associated to a more complicated surgery (see Pell and Gregory classification and type of surgery in  Table 1). This should be explained considering that patients treated under GA had an high anxiety level towards dental treatments and proceeded with third molar extraction only after developing an acute pathology of the tooth. Furthermore, the inflammatory/infective complications were much more associated to malposed and included teeth than to those normally erupted. Infact, the most frequent pathologic condition related to the avulsion in GA group was malposition in 72.2% of cases, followed by periodontitis but only in a percentage of 16%. Conversely, 73.2% of teeth extracted under LA did not need flap preparation and found carious lesion as the main cause of extraction in 47.6% of cases followed by periodontitis (21.8%) and malposition (20.3%).

As explained above, the increased surgical difficulty in the GA has to be taken in account when interpreting the odds ratio for development of IAN injures that was found to be about 16 times higher for molars extracted under GA. This value is probably overestimated because the GA group was associated to a higher operative difficulty in respect with LA group: the higher surgical difficulty, the greater risk of neurological complications. For this reason, prudence has to be applied in interpreting this result. Nonetheless, the inferior limit of the 95% confidence interval indicated that the risk was not lower than 2, denoting that neurological adverse events should be expected more frequently when treating patients under GA. The unique case of IAN involvement (permanent) in the LA group was due to an entrapment of the nerve between fused roots, suggesting that, in this patient, the adverse event was much more related to the specific anatomic condition than to surgical difficulty. This situation had been diagnosed in the pre-surgical phase and the patient was informed on the elevated risk of IAN lesion that was substantially unavoidable.

Regarding LN, no cases were found for patients treated under LA (0%) while 4 cases (2.1%) were observed for GA. These percentages are low and agree with literature when a lingual retractor is not used. However, a significant difference was observed between the two groups (Fisher exact test; *P* < 0.05), indicating that GA predisposes also to LN injures.

In conclusion, considering that possible biases as surgeon, surgeon seniority, type of surgical technique, modality of neurological assessment and follow-up, have been eliminated a priori, within the limit of the study, the null hypothesis has to be rejected since GA increases the risk of developing IAN and LN lesions. Specifically, the risk for developing IAN injures is 2 to 16 times higher in GA patients in respect with LA groups.

When treating patients that request GA, they must be informed that an increased risk of developing neurological injures is expected.
